# Antibodies in healthcare personnel following severe acute respiratory syndrome coronavirus virus 2 (SARS-CoV-2) infection

**DOI:** 10.1017/ash.2022.231

**Published:** 2022-06-15

**Authors:** Rachel E. Bosserman, Christopher W. Farnsworth, Caroline A. O’Neil, Candice Cass, Daniel Park, Claire Ballman, Meghan A. Wallace, Emily Struttmann, Henry Stewart, Olivia Arter, Kate Peacock, Victoria J. Fraser, Philip J. Budge, Margaret A. Olsen, Carey-Ann D. Burnham, Hilary M. Babcock, Jennie H. Kwon, for the CDC Prevention Epicenters

**Affiliations:** 1 Division of Infectious Diseases, Department of Medicine, Washington University School of Medicine, St. Louis, Missouri; 2 Department of Pathology and Immunology, Washington University School of Medicine, St Louis, Missouri

## Abstract

In a prospective cohort of healthcare personnel (HCP), we measured severe acute respiratory syndrome coronavirus virus 2 (SARS-CoV-2) nucleocapsid IgG antibodies after SARS-CoV-2 infection. Among 79 HCP, 68 (86%) were seropositive 14–28 days after their positive PCR test, and 54 (77%) of 70 were seropositive at the 70–180-day follow-up. Many seropositive HCP (95%) experienced an antibody decline by the second visit.

Antibodies play an important role in protection against severe acute respiratory syndrome coronavirus 2 (SARS-CoV-2), which causes coronavirus disease 2019 (COVID-19).^1^ In this study, we quantify IgG nucleocapsid (N) antibodies over time in healthcare personnel (HCP) with recent SARS-CoV-2 infection and investigated factors associated with seropositivity.

## Methods

We conducted a prospective cohort study at a large academic medical center in St Louis, Missouri. The Washington University Human Research Protection Office approved this study with documentation of informed consent.

Participants were HCP with a positive nasopharyngeal swab SARS-CoV-2 polymerase chain reaction (PCR) test. Enrollment visits, which occurred September 29, 2020, through December 23, 2020, were conducted 14-–28 days after the positive PCR test. Follow-up visits were conducted 70–180 days after the positive test (between December 7, 2020, and April 13, 2021). At both visits a blood specimen was obtained, and participants completed a survey. Further details pertaining to participants and analyses are described in the Supplementary Material.

## Results

In total, 113 HCP with a positive SARS-CoV-2 PCR provided informed consent; 79 completed an enrollment visit; and 70 completed a follow-up visit. Supplementary Table 1 shows characteristics of the cohort and potential occupational and nonoccupational risk factors in the 30 days prior to the enrollment visit. Among the 79 enrolled HCP, 81% were women, 90% were white, and the median age was 35 years (interquartile range [IQR], 28–46).

Overall, 68 HCP (86%) were seropositive for IgG N-antibodies at the enrollment visit, which occurred a median of 24 days (IQR, 20.5–25) after the positive SARS-CoV-2 PCR test. No demographic factors were associated with seropositivity (Table 1).

**Table 1.** Bivariate Risk Factors for a Positive SARS-CoV-2 Antibody Test Result at Enrollment (N = 79)

Variable	Reactive Antibody Test Result (n=68), No. (%)	Nonreactive Antibody Test Result (n=11). No. (%)	*P* Value
**SARS-CoV-2 Testing**			
Days from positive SARS-CoV-2 PCR test, median d (IQR)	24 (20.5–25)	22 (17–24)	.07
**Demographics**			
Age ≥50 y	14 (20.6)	1 (9.1)	.68
White race	61 (89.7)	10 (90.9)	1.00
Hispanic ethnicity	2 (3.0)	1 (9.1)	.37
Female sex	54 (79.4)	10 (90.9)	.68
Patient care job role	43 (63.2)	7 (63.6)	1.00
Working on campus	51 (75.0)	9 (81.8)	.28
Comorbidities			
Seasonal allergies	28 (41.2)	5 (45.5)	1.00
Obesity	12 (17.7)	2 (18.2)	1.00
Other^a^	19 (27.9)	4 (36.4)	.72
**Symptoms in the 14 d prior to enrollment antibody testing**			
Any symptom	46 (67.6)	5 (45.4)	.18
Fatigue	33 (50.0)	3 (27.3)	.33
Headache	32 (47.1)	2 (18.2)	.10
Cough	29 (43.3)	4 (36.4)	.75
Congestion or runny nose	23 (34.3)	4 (36.4)	1.00
Muscle or body aches	23 (33.8)	2 (18.2)	.49
New loss of sense of taste or smell	22 (32.4)	1 (9.1)	.16
Shortness of breath	14 (20.9)	1 (9.1)	.68
Fever or chills	12 (18.2)	1 (9.1)	.68
GI symptoms	10 (14.7)	2 (18.2)	.67
Sore throat	7 (10.4)	2 (18.2)	.60
Other^b^	6 (8.8)	0 (0.0)	
>1 symptom	39 (57.4)	4 (36.4)	.21
No. of symptoms (IQR)^c^	4 (2–6)	2 (2–8)	.30
**Ongoing symptoms at time of enrollment survey**			.31
Yes	14 (20.6)	2 (18.2)	
No	32 (47.1)	3 (27.3)	
Never experienced symptoms	22 (32.3)	6 (54.5)	
**Sought medical care for symptoms^c^**			1.00
Sought medical care	14 (20.6)	1 (9.1)	
Did not seek medical care	32 (47.1)	4 (36.4)	

a Other comorbidities (reported by <5 participants each) include asthma, cerebrovascular disease, eosinophilic esophagitis, epilepsy, Graves’ disease, hearing loss, hypertension, hypothyroidism, liver disease, lung disease, migraine, pregnancy, psoriasis, smoking, and use of corticosteroids or other immunosuppressive drugs.

b Other symptoms include discolored sputum, dizziness, sinus drainage, sinus pressure, skin hypersensitivity, and rash.

c Among HCP who reported having symptoms.

Of 79 HCP, 51 (65%) reported having symptoms within 14 days of the enrollment antibody test; none developed severe COVID-19 requiring hospitalization. No symptoms or comorbidities were associated with seropositivity (Table 1). There was no difference in median antibody signals for those who reported symptoms compared to those without symptoms: 5.51 index specimen/calibrator (S/C) versus 4.28 S/C (*P* = .28) (Supplementary Fig. 1A)

In total, 70 HCP completed the 70–180-day follow-up visit, which occurred a median of 84 days (IQR, 77–92) after the positive PCR test. Among them, 54 HCP (77%) were seropositive at follow-up (Supplementary Table 2). At follow-up, 16 HCP (23%) reported ongoing symptoms (Supplementary Table 3); 10 (63%) reported diminished taste and/or smell. There was no difference in median IgG N-antibody signal nor median SARS-CoV-2 PCR Ct values between HCP who reported ongoing symptoms and those who did not (Supplementary Fig. 1B and Supplementary Fig. 2).

**Fig. 1.**
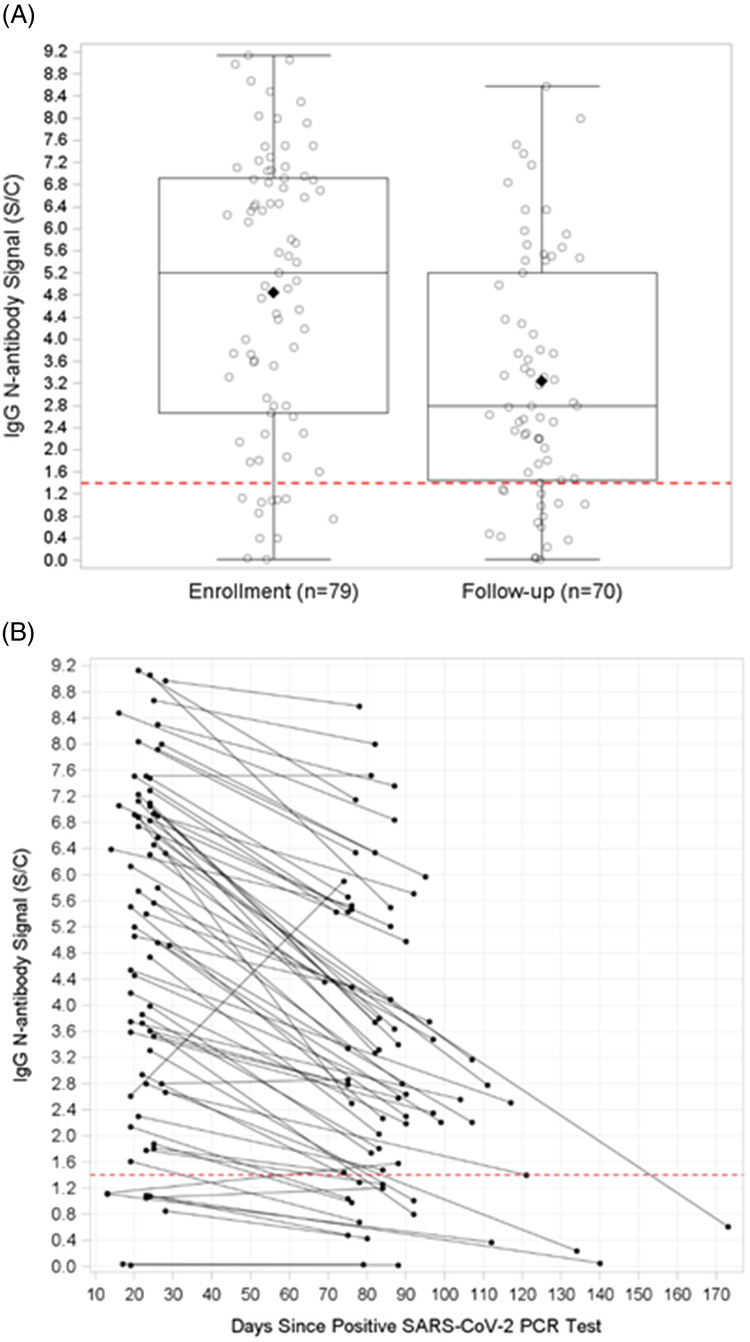
IgG N-antibody signal at enrollment and follow-up. (A) Comparison of IgG N-antibody signal at the enrollment and follow-up visits. The median IgG N-antibody signal at the enrollment visit was 5.20 (IQR, 2.67–6.92) compared to the median IgG N-antibody signal at follow-up which was 2.79 (IQR, 1.44–5.21). IgG N-antibody signal was significantly decreased at the follow-up visit compared to the enrollment visit (Wilcoxon signed rank test *P* < .001). The solid diamonds represent the mean antibody signal at enrollment and follow-up, 4.84 and 3.20 index specimen/calibrator, respectively. (B) IgG N-antibody signals over time for each participant with antibody test results from both study visits (n = 70). The dotted line represents the seropositivity threshold.

Median antibody signal decreased between enrollment (median, 5.20 S/C) and follow-up visit (median, 2.78 S/C; Wilcoxon signed rank *P* < .001) (Fig. 1A). Figure 1B shows individual-level IgG N-antibody signals over time. Of the 62 HCP who were seropositive at enrollment and who completed follow-up visits, 59 (95%) had decreased antibody signal at follow-up (average decrease, 42.4%; Wilcoxon signed rank *P* < .001), and 9 (15%) experienced seroreversion (ie, changed from seropositive to seronegative).

Of the 11 HCP who were initially seronegative, 8 completed follow-up visits. One HCP seroconverted between enrollment and follow-up. In addition, 10 HCP reported potential COVID-19 exposures that occurred between study visits; however, all 10 HCP had decreased antibody signal between enrollment visits (median, 5.48 S/C; IQR, 3.59–6.33) and follow-up visits (median, 3.06 S/C; IQR, 2.21–4.36; Wilcoxon signed rank *P* = .002).

We detected no correlation between Ct values and IgG N-antibody signal at enrollment (Supplementary Figure 3A, Spearman ρ = −0.149; *P* = .26). However, there was a weak negative correlation between Ct value and IgG N-antibody signal at follow-up (Spearman ρ = −0.314; *P* = .022) (Supplementary Fig. 3B). Seropositive HCP had a lower median Ct than seronegative HCP at both study time points, although the difference was not significant (Supplementary Fig. 4A and 4B).

## Discussion

The objective of this study was to measure antinucleocapsid IgG signal over time in HCP with a recent positive SARS-CoV-2 PCR test. Overall, 86% of HCP were seropositive 14–28 days after their positive PCR test, and 77% were seropositive at the 70–180-day follow-up visit. For most seropositive HCP, IgG N-antibody signal decreased between study visits. Our findings align with prior reports of 81%–94% seropositivity for N-or Spike (S)-antibodies following SARS-CoV-2 infection.^2–4^ All enrollment visits took place before SARS-CoV-2 variants of interest were recognized in December 2020. Future work is needed to understand whether infection with SARS-CoV-2 variants elicit more or less robust N-antibody responses.

More severe COVID-19 has been associated with higher viral loads, approximated by lower Ct values,^5^ and higher peak antibody levels.^3^ In the current study, we found no correlation between Ct value and N-antibody signal 14–28 days after the positive PCR test; however, having no severe COVID-19 cases limited our analysis. We found a weak inverse correlation between Ct values and N-antibody signal at the 70–180-day follow-up, similar to the correlation previously reported between S-antibody signal, measured 10–68 days after symptom onset, and mean Ct values of 117 SARS-CoV-2 PCR–positive participants.^2^ However, correlation testing often overestimates statistically significant but clinically weak correlations. Although biologically plausible, the lack of a standard time from exposure or symptoms to PCR testing is a limitation of our study.

In a study involving >2,000 first responders, S-antibody seropositivity was associated with being Black/Non-Hispanic, severe obesity, and reporting more symptoms, whereas immunosuppression was associated with seronegativity.^4^ We found no associations with N-antibody seropositivity; however, our small cohort lacked diversity (eg, immunosuppressed, elderly) for detecting potential risk factors.

In our study, 10 HCP reported COVID-19 exposures between study visits yet had decreased antibody signal at follow-up. These events may not have been true exposures or reinfection may not have occurred.

Moreover, 16 HCP (23%) had ongoing symptoms at follow-up, consistent with postacute sequelae of COVID-19 (PASC).^6^ In a previous study of PCR-positive mobile health application users, 13% of participants reported symptoms lasting ≥ 28 days.^7^ In our cohort, comprised largely of young, healthy HCP, loss of taste or smell was the most common ongoing symptom. Similar to our findings, Pereira et al^8^ also found no association between antibody levels or Ct values and having PASC conditions. How PASC impacts the immune response and vice versa remains unknown.

Our study had several limitations. The small cohort resulted in limited demographic diversity. Symptoms were self-reported and were restricted to the 14 days prior to antibody testing, which may have led to underreporting of symptoms. The timing of testing and other factors may have influenced both antibody and Ct results. To mitigate these effects, we limited antibody testing to defined windows relative to the PCR test and did not compare PCR Ct values across testing platforms. However, the Ct values may also be impacted by viral kinetics, specimen collection technique, and specimen transport.^9^ Additionally, the nature of the exposure and symptoms at the time of the initial PCR test were unavailable.

A strength of this study is the detailed survey data linked by date to antibody testing at 2 time points. Our results demonstrate a varied IgG N-antibody response following SARS-CoV-2 infection. The long-term clinical relevance of antibody testing is still being determined, as immunological memory cells can persist, even as circulating antibodies taper over time.^10^ Determining the persistence of antibodies and the correlation of antibodies with protection from reinfection is important for protecting HCP who may experience continual exposure to SARS-CoV-2. Hospitals could periodically monitor antibodies in HCP, particularly those in high-risk settings, once a threshold of protection is identified.

## Supplementary material

For supplementary material accompanying this paper visit https://doi.org/10.1017/ash.2022.231. click here to view supplementary material
