# Supplementary material for: Antibodies in healthcare personnel following severe acute respiratory syndrome coronavirus virus 2 (SARS-CoV-2) infection
Source: Antimicrob Steward Healthc Epidemiol. 2022 Jun 15;2(1):e93. doi: 10.1017/ash.2022.231 (PMC9726486; doi:10.1017/ash.2022.231)
Supplement: Supplementary file 1 [file S2732494X22002315sup001.doc]

**SUPPLEMENTARY MATERIAL**

**SUPPLEMENTARY METHODS**

**Study Participants**

HCP ≥ 18 years of age who were employed at Washington University School of Medicine, Barnes-Jewish Hospital, or St. Louis Children’s Hospital, and who had a positive SARS-CoV-2 PCR test from a nasopharyngeal swab within the previous 28 days were eligible. Participants were identified using records from an Occupational Health COVID-19 Employee Call Center and were contacted within 18 days of the PCR test date. At the time of the study, Occupational Health tested HCP who were symptomatic or a part of contact tracing/exposure investigations.

**Specimen Collection and Laboratory Testing**

Blood specimens were drawn into a 10mL K_2_ EDTA tube and maintained at room temperature for up to 8 hours before refrigeration. Specimens were centrifuged and plasma aliquots stored for up to 4 days at 4°C prior to analysis.

*Antibody Testing*. Anti-N IgG was detected in plasma samples using the Abbott SARS-CoV-2 IgG assay (Abbott Laboratories, Abbott Park, IL) on the Abbott Architect i2000, with testing performed according to the manufacturer’s instructions. Results were calibrated with a relative light unit calibrator, used to calculate a ratio of specimen absorbance to calibrator absorbance. Samples with an Index Specimen/Calibrator (S/C) value of 1.4 or greater were interpreted as positive.

*SARS-CoV-2 RT-PCR.* Records from the positive PCR test were obtained, including cycle threshold (C_T_) values, when available, for participants who received testing BJC/WUSM OH performed at the BJH clinical laboratory. Six HCP received SARS-CoV-2 testing elsewhere. Due to supply chain shortages, the laboratory used a variety of testing platforms during the study period. Seventy-nine percent (58/73) of the NP swabs with C_T_ values available were tested with the Roche cobas® SARS-CoV-2 assay (Roche Diagnostics, Indianapolis, IN), 11% (8/73) with the Quidel Lyra SARS-CoV-2 assay (Quidel, San Diego, CA), and 9.6% (7/73) with the Diasorin Simplexa COVID-19 Direct assay (DiaSorin Molecular, Cypress, CA). To avoid measurement error, only Roche C_T_ values were used in the data analyses.^1^ The Roche assay reports two C_T_ values, as it detects two SARS-CoV-2 targets (*orf1ab* and *E*). The lowest Roche C_T_ value was used for the purpose of analyses. For the one participant with C_T_ values near the limit of detection (0/37.67), the C_T_ value for the positive target (*E* gene: 37.67) was used.

**Statistical Analyses**

Fisher’s exact tests were used to examine associations between N-antibody test results and HCP characteristics, SARS-CoV-2 exposure history, and symptoms. Wilcoxon Rank Sum tests and Wilcoxon Signed Rank tests were used to evaluate differences in median N-antibody signal or C_T_ values among groups. Spearman’s correlation was used to determine the relationship between C_T_ values and N-antibody signal. Statistical analyses were performed in SAS version 9.4 (Cary, NC), with *p* < 0.05 considered statistically significant.

**TABLES AND FIGURES**

**TABLES**

**Supplementary Table 1. Cohort Characteristics**

| **Enrollment survey questions** | **Frequency**  **N = 79 (%)** |
| --- | --- |
| **Demographics** | |
| Age (Median, [IQR]) | 35 (28, 46) |
| Age, categorical |  |
| 20-29 years | 23 (29.1) |
| 30-39 years | 24 (30.4) |
| 40-49 years | 17 (21.5) |
| 50-59 years | 11 (13.9) |
| 60 + years | 4 (5.1) |
| Female | 64 (81.0) |
| Race^a^ |  |
| Asian | 4 (5.1) |
| Black | 3 (3.8) |
| White | 71 (89.9) |
| Other | 1 (1.3) |
| Hispanic ethnicity | 3 (3.8) |
| **Employment Information** | |
| Department |  |
| Medical/Surgical Wards/Specialties | 43 (54.4) |
| ICU | 7 (8.9) |
| Emergency Services | 7 (8.9) |
| Pediatrics | 4 (5.1) |
| Pathology/Pharmacy/Radiology | 4 (5.1) |
| Laboratory | 2 (2.5) |
| Other^b^ | 12 (15.2) |
| Job role |  |
| Direct patient care role^c^ | 41 (51.9) |
| Other patient care role^d^ | 14 (17.7) |
| Non-patient care role^e^ | 23 (29.1) |
| Missing | 1 (1.3) |
| Commute method^a^ |  |
| Drive a car alone | 73 (92.4) |
| Drive a car with others | 3 (3.8) |
| Other^f^ | 2 (2.5) |
| Missing | 1 (1.3) |
| **Occupational risk factors** | |
| Known, specific COVID-19 exposure at work | 13 (16.7) |
| Contact with known or suspected COVID-19 patients at work |  |
| More than half the time | 10 (12.7) |
| Less than half the time | 41 (51.9) |
| Never | 25 (31.6) |
| Missing | 3 (3.8) |
| Practice social distancing when at work and not involved in immediate patient care |  |
| More than half the time | 50 (63.3) |
| Less than half the time | 27 (34.2) |
| Never | 1 (1.3) |
| Missing | 1 (1.3) |
| Wear a face mask while at work |  |
| More than half the time | 78 (98.7) |
| Missing | 1 (1.3) |
| Wear a cloth mask at work^a,g^ | 16 (20.3) |
| Wear a surgical/isolation (disposable) mask at work^a,g^ | 70 (88.6) |
| Wear a N-95 mask at work^a,g^ | 9 (11.4) |
| **Non-occupational risk factors** | |
| Known, specific COVID-19 exposure outside of work | 22 (27.9) |
| Practice social distancing when in public |  |
| More than half the time | 78 (98.7) |
| Less than half the time | 1 (1.3) |
| Wear a face mask while in public |  |
| More than half the time | 79 (100.0) |
| Traveled in the past 30 days | 10 (12.7) |
| Attended a gathering in the past 30 days |  |
| Restaurant | 28 (35.4) |
| Other large gathering^h^ | 28 (35.4) |
| None | 31 (39.2) |
| Visited a public location in the past 30 days |  |
| Medical office | 22 (27.8) |
| Store | 75 (94.9) |
| Other^i^ | 23 (29.1) |
| None | 3 (3.8) |
|  |  |
| **SARS-CoV-2 PCR Testing** | |
| Days from positive SARS-CoV-2 PCR test (Median, [IQR]) 23 (20, 25) | |
| Roche C_T_ value (Median, [IQR])^j^ | 19.5 (17.5, 24.2) |

^a^ Participants could select multiple responses for this question.

^b^Other departments include: Case management, food and nutrition, operations and facilities management, patient transportation, research.

^c^Provider with direct patient care roles include: Advanced practice nurse, nurse, nurse practitioner, physician, physician’s assistant.

^d^ Other patient care roles include: Medical student, nurse assistant, paramedic, patient care technician, perfusionist, physical therapist, occupational therapist.

^e^ Non-patient care roles include: Administration, case manager, dietician, dining services personnel, laboratory personnel, pharmacist, research personnel, social worker.

^f^ Other transportation methods include: Bike, train.

^g^ During the study period, PPE recommendations varied by job and location. Cloth masks were recommended for non-clinical staff, surgical masks were recommended in most clinical spaces, and N95s were recommended in COVID ICUs and during aerosol-generating procedures.

^h^Other large gatherings include: Bar, funeral, gatherings of family or friends, outdoor sporting event, religious service, school, wedding. Large gathering was defined as an event having >10 people outside of their household.

^i^Other locations include: Bank, DMV, dry cleaner, hair salon, laundromat, library, post office, vet.

^j^Among 58 HCP with Roche C_T_ values

**Supplementary Table 2. Bivariate risk factors for a reactive Anti-N IgG** **antibody result at follow-up (N=70)**

|  | **Reactive**  **antibody test result**  **n=54 (%)** | **Non-reactive antibody test result**  **n=16 (%)** | **p-value** |
| --- | --- | --- | --- |
| **Demographics** | | | |
| Age, 50 years or older | 14 (25.9) | 1 (6.2) | 0.16 |
| White race | 48 (88.9) | 14 (87.5) | 1.00 |
| Hispanic ethnicity | 2 (3.8) | 1 (6.2) | 0.56 |
| Female sex | 45 (83.3) | 12 (75.0) | 0.48 |
| Patient care job role | 36 (66.7) | 8 (50.0) | 0.25 |
| Working on campus | 46 (85.2) | 14 (87.5) | 1.00 |
| Comorbidities |  |  |  |
| Seasonal allergies | 22 (40.7) | 7 (43.8) | 1.00 |
| Obesity | 11 (20.4) | 3 (18.8) | 1.00 |
| Other^a^ | 15 (27.8) | 6 (37.5) | 0.54 |
| **Symptoms** | | | |
| Ongoing Covid-19 symptoms | 11 (20.4) | 5 (31.2) | 0.50 |
| Duration of COVID-19 symptoms |  |  | 0.046 |
| Less than 7 days | 10 (18.5) | 2 (12.5) |  |
| 7 – 13 days | 14 (25.9) | 3 (18.8) |  |
| 14 – 20 days | 8 (14.8) | 2 (12.5) |  |
| 21 – 27 days | 6 (11.1) | 0 (0.0) |  |
| 1 – 2 months | 2 (3.7) | 2 (12.5) |  |
| More than 2 months | 12 (22.2) | 2 (12.5) |  |
| Never had symptoms | 1 (1.8) | 1 (6.3) |  |
| Not sure / Missing | 1 (1.8) | 4 (25.0) |  |
| **Exposures since enrollment survey was completed** | | | |
| COVID-19 exposure at work |  |  | 0.07 |
| Yes | 5 (9.3) | 1 (6.2) |  |
| No | 49 (90.7) | 13 (81.2) |  |
| Missing | 0 (0.0) | 2 (12.5) |  |
| COVID-19 exposure to household member |  |  | 0.05 |
| Yes | 1 (1.8) | 0 (0.0) |  |
| No | 53 (98.1) | 14 (87.5) |  |
| Missing | 0 (0.0) | 2 (12.5) |  |
| COVID-19 exposure outside of work or household |  |  | 0.06 |
| Yes | 3 (5.6) | 0 (0.0) |  |
| No | 51 (94.4) | 14 (87.5) |  |
| Missing | 0 (0.0) | 2 (12.5) |  |

**^a^** Other comorbidities (reported by <5 participants each) include: Asthma, cerebrovascular disease, eosinophilic esophagitis, epilepsy, Graves’ disease, hearing loss, hypertension, hypothyroidism, liver disease, lung disease, migraine, pregnancy, psoriasis, smoking, use of corticosteroids or other immunosuppressive drugs

**Supplementary Table 3. Ongoing Symptoms Reported at Follow-up in 16 HCP**

| **Symptom** | **Frequency**  **N=16 (%)** |
| --- | --- |
| Loss of sense of taste or smell | 10 (63%) |
| Persistent fatigue | 3 (19%) |
| Shortness of breath | 2 (13%) |
| Headaches | 2 (13%) |
| “Brain fog” | 2 (13%) |
| Joint pain | 1 (6.3%) |
| Diarrhea | 1 (6.3%) |
| Cough | 1 (6.3%) |

**FIGURE LEGENDS**

**Supplementary Figure 1. IgG N-antibody signal stratified by reported symptoms. A)** Antibody signals were not different between those who reported being symptomatic and those who reported being asymptomatic at enrollment (Wilcoxon Rank Sum Test, p=0.28). Asymptomatic HCP had a median signal of 4.28 (IQR: 2.04-6.87) compared to the median antibody level of symptomatic HCP (5.51, [IQR: 2.8-6.94]). **B)** Antibody signals were not different between those reported ongoing symptoms at follow-up and those who had no ongoing symptoms (Wilcoxon Rank Sum Test, p=0.15). For those with no ongoing symptoms, the median signal was 3.30 S/C (IQR: 1.58-5.47). For those with ongoing symptoms, the median signal was 2.24 (IQR: 1.25-2.98). The dotted line represents the seropositivity threshold.

**Supplementary Figure 2.** **Roche C_T_ values stratified by symptoms reported in the follow-up survey.** Roche RT-PCR C_T_ values were compared among HCP who reported no ongoing symptoms versus those with ongoing symptoms at follow-up**.** The median C_T_ value for those with no ongoing symptoms was 18.8 (IQR: 17.5-23.5) compared to the median C_T_ value for those with ongoing symptoms, which was 24.8 (IQR: 16.7-27.0), (Wilcoxon Rank Sum Test, p=0.20). The lowest reported Roche C_T_ value was used in the analysis.

**Supplementary Figure 3. Relationship between C_T_ values and IgG N-antibody signal.** **A)** Roche RT-PCR C_T_ values do not significantly correlate with IgG N-antibody signal measured at enrollment, 14-28 days after the positive SARS-CoV-2 PCR test. Spearman correlation coefficient = -0.149 (p=0.26). **B)** Roche RT-PCR C_T_ values significantly correlate with IgG N-antibody signal measured at follow-up, 70-180 days after the positive SARS-CoV-2 PCR test. Spearman correlation coefficient = -0.314 (p=0.022). The lowest reported Roche C_T_ value was used in both analyses. The dotted line depicts the seropositivity threshold.

**Supplementary Figure 4. Roche C_T_ values stratified by IgG N-antibody test result. . A)** Roche C_T_ values were compared among HCP who had reactive and nonreactive N-antibody results at enrollment**.** The median C_T_ value for non-reactive HCP was 22.5 (IQR: 18.3-26.3) compared to the median C_T_ value for reactive HCP which was 19.2 (IQR: 17.5-23.9), (Wilcoxon Rank Sum Test, p=0.48). **B)** Roche C_T_ values were compared among HCP who had reactive and nonreactive N-antibody results at follow-up. The median C_T_ value for non-reactive HCP was 23.5 (IQR: 21.7-26.3) compared to the median C_T_ value for reactive HCP which was 18.8 (IQR: 17.2-23.9), (Wilcoxon Rank Sum Test, p=0.12). The lowest reported Roche C_T_ value was used in both analyses.

**FIGURES**

**Supplementary Figure 1. IgG N-antibody signal stratified by reported symptoms.**


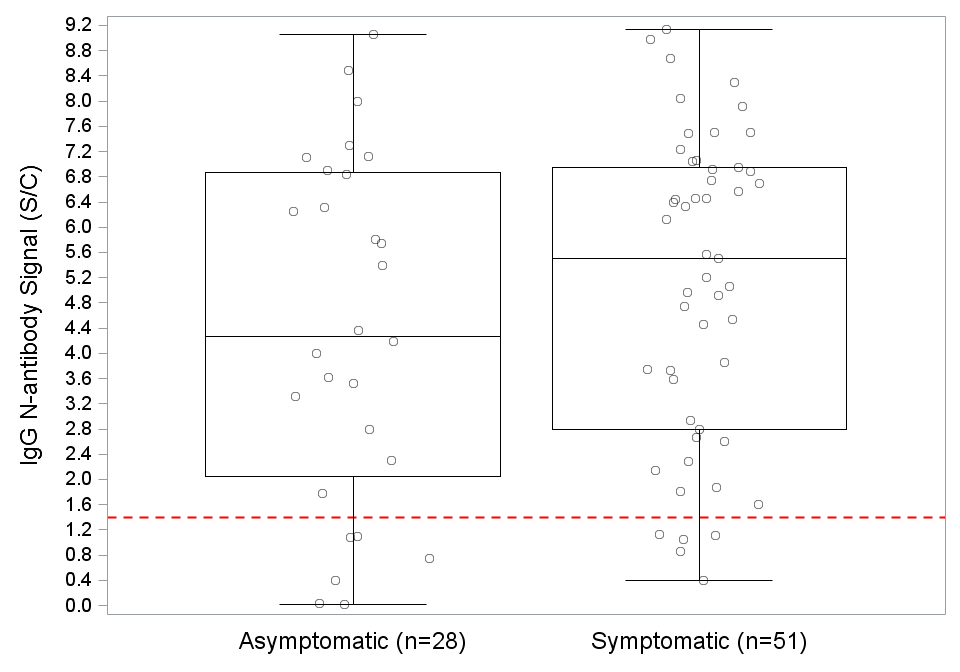


**
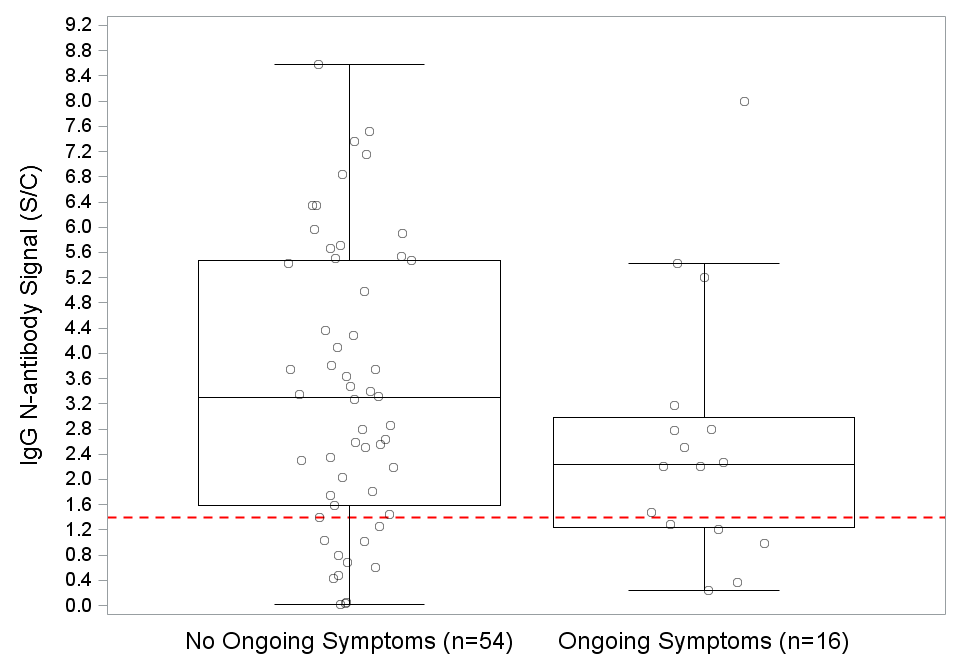
**

**Supplementary Figure 2.** **Roche C_T_ values stratified by symptoms reported in the follow-up survey.**


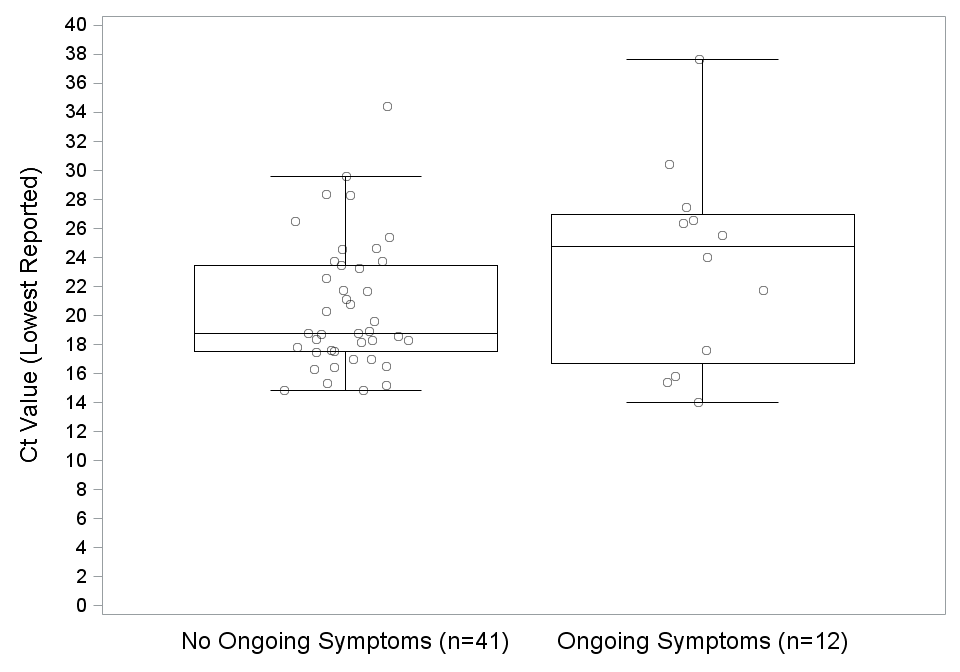


**Supplementary Figure 3. Relationship between C_T_ values and IgG N-antibody signal.**

**A.**


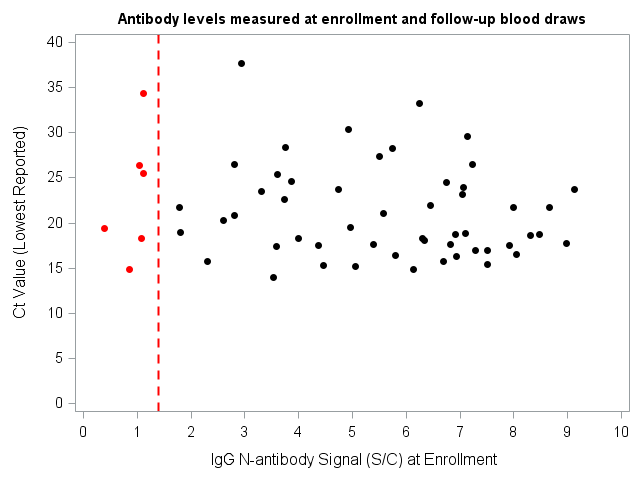


**B.**

**
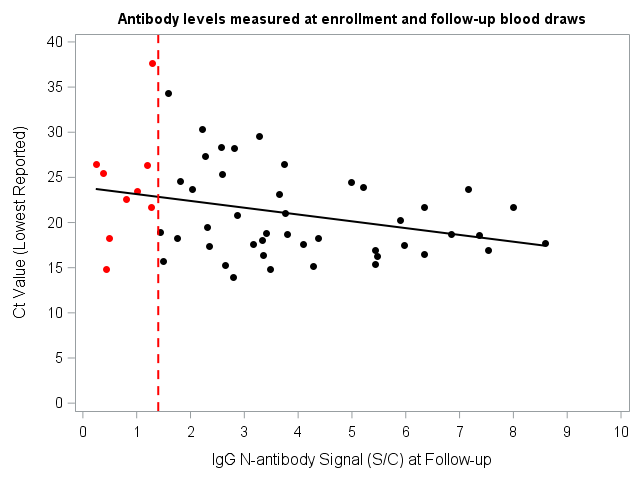
**

**Supplementary Figure 4. Roche C_T_ values stratified by IgG N-antibody test result.**

**A.**


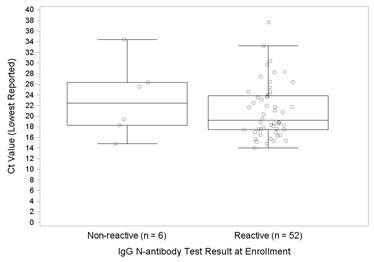


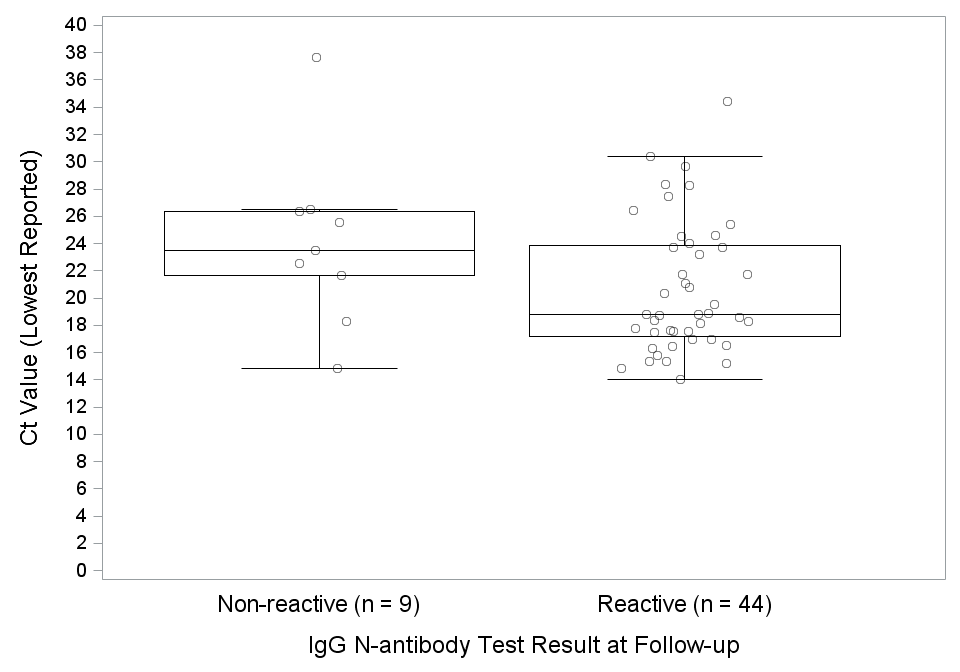
**B.**

**REFERENCES**

1. Rhoads D, Peaper DR, She RC, et al. College of American Pathologists (CAP) Microbiology Committee Perspective: Caution Must Be Used in Interpreting the Cycle Threshold (Ct) Value*. Clin Infect Di*s 2021;72:e685-e686.
